# Evaluating patients’ perceptions regarding generic medicines in Jordan

**DOI:** 10.1186/2052-3211-6-3

**Published:** 2013-06-13

**Authors:** Faris El-Dahiyat, Reem Kayyali

**Affiliations:** 1 Pharmacy Department, Kingston University, Penrhyn Road, Kingston upon Thames, KT1 2EE, United Kingdom

**Keywords:** Generic medicines, Generic substitution, Cost, Patients, Perception, Policy

## Abstract

**Objective:**

The aim of this study was to explore Jordanian patients’ perceptions toward generic medicines and to evaluate their opinions regarding generic substitution.

**Method:**

A cross-sectional descriptive study involving Jordanian patients was undertaken, using a self-administrated anonymous questionnaire. The response rate was 80% (n=400/500).

**Results:**

The study showed that cost of medicines is high according to 83% of the patients. Most patients (92%) preferred to be prescribed the cheapest medicine. Majority of patients (79%) believed that cost should be considered before a drug is prescribed. Most patients (78%) accepted generic substitution and believed that it can provide significant saving. Surveyed patients (78%) agreed that they should have the option of choosing between generic and originator and 74% believed that physicians should give them that choice. These results showed a significant statistical correlation with the monthly income of the patient, percentage cost they pay and number of medicines prescribed (P<0.05).

**Conclusion:**

The high cost of medicines in Jordan is believed to be the main driver for choosing generic medicines Furthermore; patients have positive attitudes towards generic medicines. The involvement of patients in the treatment decision would result in more adherence and improvement in health. The insights gained from patients in this study will be useful to health organisations and policy makers to design a robust generic policy to use medicines cost-effectively in Jordan.

## Introduction

Generic substitution is the practice of switching from a prescribed originator medicine to an interchangeable generic medicine containing the same active ingredient, dosage form, strength at the time of dispensing [1]. Generic medicines are generally marketed under the non-proprietary name or could be marketed as branded generics [2], as in the case of Jordan where 97% of generic medicines are branded [3].

The generic substitutions practice is increasingly encouraged by health authorities throughout the world [4], and Jordan is no exception. In 2002, a circular from the Jordanian Ministry of Health required doctors in public hospitals and health clinics to prescribe generically. However, if a brand name is prescribed, the patient gets the formulary drug anyway, unless their physician builds a case and receives special permission to have the brand name dispensed. Furthermore, private health insurance companies encourage doctors to prescribe the lowest priced generic [5]. Nevertheless, under the current Jordanian legislation, pharmacists are not permitted to make any change or substitution to prescriptions, unless the pharmacist contacts the prescriber and requests permission for the prescribed originator medicine to be substituted to an alternative generic medicine [6].

The use of cheaper generic medicines is often promoted as a measure to reduce the health care expenditure on pharmaceutical products, and provide savings to patients as well as governments. Generally, the generic medicines are 20-90% less expensive than the innovator medicines [7].

It has been estimated that €25 billion (more than $30 billion) is the annual save made by European patients and health care systems for using generic medicines [7]. Furthermore, it was reported that the use of generic medicines saved American patients, taxpayers, federal and state governments and other payers $193 billion in 2011 alone, and around $1.07 trillion over the period 2002 to 2011 [8]. A World Health Organisation (WHO) study carried out in several developing countries including Jordan estimated an average saving of 9% to 89% could be made by an individual country from substituting some originator brands to lowest-priced generics [9]. In addition the report stipulated that the saving in Jordan could be 56% if only 11 originator medicines switched to lowest available generics [9].

Despite the financial benefits from using generic medicines, there are still debates regarding generic substitution by patients as well as prescribers, with regards to its effect on patients’ clinical outcomes [10-12]. A German study found that half of the primary care patients are sceptical about generic substitution, and 13% of the patients reported that they had experienced new adverse reactions [13]. On the other hand, another study revealed that 61% of the Slovakian patients had positive views regarding generic medicines [14]. The views in the former study were expressed by patients who were more than 60 years of age, chronically ill, and/or without higher education. In the latter study the respondents were predominantly aged 30 years or younger. This indicates that patients’ socio demographic characteristics such as educational level, income and age may influence people’s opinions of generic drugs [15].

Other factors that may influence patients’ attitudes towards generic medicines are believed to be the physicians’ prescribing behaviour and their preferences for particular originator brand or their bias against generics [16]. Moreover, the information given by a prescribing physician on generic substitution was also found to be a main driver that influences patients’ beliefs about generic medicines and their consumptions [13,17]. Previous studies showed that physicians and pharmacists play an important role when patients choose between branded or generic drugs [18-20]. Therefore efforts to promote generic substitutions practice should be targeted first and foremost at time of prescribing as well as dispensing [21].

Although patient perceptions may play an important role in medication selection, previous research revealed that patients often do not communicate with their physicians about their medicines preference and cost of medications. Furthermore, several studies found that the high out of pocket-costs can be a significant obstacle to medical adherence with prescription medication regimens [22-24]. However, patients can still request generic medications at the point of the clinical encounter or at the time of dispensing of the medication at the pharmacy [25]. In Jordan, over 80% of the cost of medicines purchased by the public is funded through out-of pocket payments [26].

Patient willingness to accept a generic medicine is a core requirement to facilitate the uptake of generic medicines [27,28]. However, there is lack of studies which investigated Jordanian patients’ perceptions about generic medicines, their opinions regarding costs of medicines, and their acceptance of generic substitution.

The aim of this study was to assess the understanding and attitude of Jordanian patients’ towards generic medicines, their opinions about the cost of medicines in general, and to evaluate their perceptions about generic substitution. The findings from this study would provide a baseline data for establishing a robust generic medicine policy in Jordan.

## Methods

This was a cross sectional study where a questionnaire was used to collect data from Jordanian patients whom were targeted by visiting private and public clinics, private and public hospitals, community pharmacies and The National Centre for Diabetes, Endocrinology & Genetics in Jordan. One of the researchers was available on site if the responders need any clarification at the time of the study.

The questionnaire was tested for face and content validity by two experts. It was further revised after pilot testing with 20 patients. Patients were given an information sheet translated to Arabic language by certified translator that explained the research. The questionnaire was also translated to Arabic language by a certified translator.

The questionnaire used consisted of three sections. The first section gave a simple definition of originator and generic medicines with examples. The second section evaluated the preferred prescribed medicines and the perceptions regarding originator to generic substitution and the costs of medicines in Jordan. The last section characterised the respondent demographics.

The responses were framed in four point likert scale (1 = strongly disagree, 2 = disagree, 3 = agree and 4 = strongly agree) questions.

In this study, the sample population was Jordanian patients with chronic medical conditions. From the 500 questionnaires which were distributed, 400 questionnaires were completed and included in this study which gives a response rate of 80%. The participation of patients approached was strictly voluntary and their informed consent was obtained. Anonymity of respondents was preserved in the study, as names of participants were not included.

Data was collected from 15th June 2012 to 26th August 2012. All the collected data were entered into PASW® 18.0 for descriptive analysis using descriptive statistics techniques such as frequency and cross-tabulation and inferential statistics using chi square tests. This study was approved by the Research Ethics Committee of Kingston University, London.

## Results

### Demographic characteristics of responding patients

A total of 400 responses were received, with a response rate of 80%, the basic demographic of the responding patients is summarised in Table 1. The sample was almost equally distributed between male (142, 48.3%) and female (152, 51.7%). The majority of the respondents’ monthly income was less than 500 JD (59.25%) and holding bachelor degree (42.5%). The respondents mostly pay full cost of their prescription (63.25%) and have more than 6 medicines in their prescription (78.5%) (Table 1).

**Table 1:** Demographics and characteristics of the responders

***Characteristic***	***N (% )***
***The monthly income***	
Less than 500 JD	237 (59.25)
501-1000 JD	84 (21.00)
More than 1001 JD	79 (19.75)
***Educational level***	
Post graduate	79 (19.75)
Bachelor degree	170 (42.50)
College	62 (15.50)
High school	89 (22.25)
***Percentage paid from the prescription cost***	
Do not pay at all	81 (20.25)
Pay only a percentage	66 (16.50)
Pay full cost	253 (63.25)
***No. of medicines in the prescription***	
1-3	29 (7.25)
4-6	57 (14.25)
More than 6	314 (78.50)
***Chronic Medical condition***	
Cardio-vascular diseases	122 (30.50)
Endocrine diseases	138 (34.50)
Respiratory diseases	95 (23.75)
Other chronic diseases	45 (11.25)
***General health Status***	
Poor	18 (4.50)
Fair	64 (16.00)
Good	142 (35.50)
Very good	121 (30.25)
Excellent	55 (13.75)

### Patients’ views on preferred physicians’ communications

When assessing the patients’ views on preferred communication with physicians, they predominantly agreed that the physician should ask them about their medicines preference (74%, n = 296) (Table 2). There was a significant correlation (P < 0.05) between patients’ education level and whether or not they prefer to be asked about their medicines preferences (Table 3). As the education level of the responders increased their preferences to be consulted about their medicine choices increased.

**Table 2:** Patients’ responses to four point likert scale questions exploring their perception about generic medicines

**Question**	**Survey questions/Statement**	**Frequency (%)**			
		**Strongly disagree**	**Disagree**	**Agree**	**Strongly agree**
1	Physicians should ask patients about their medicines preference.	29 (7.25)	75 (18.75)	174 (43.5)	122 (30.5)
2	Patients should have the option of choosing between generic and originator.	33 (8.25)	55 (13.75)	221 (55.25)	91 (22.75)
3	I don’t mind the pharmacist substituting the medicine I was prescribed to a cheaper equivalent one.	8 (2.00)	92 (23.00)	235(58.75)	65 (16.25)
4	I don’t mind my prescribed medicines to be substituted from originator to generic. (e.g. Panadol to Revanin).	6 (1.50)	82 (20.50)	228 (57.00)	84 (21.00)
5	My medicines should only be substituted from originator to generic if I request. (e.g. Panadol to Revanin).	69 (17.25)	77 (19.25)	141 (35.25)	113 (28.25)
6	I don’t mind the pharmacist substituting my prescribed medicine to an equivalent locally produced one.	3 (0.75)	84 (21.00)	204 (51.00)	109 (27.25)
7	I prefer to be prescribed locally produced medicines.	3 (0.75)	97 (24.25)	178 (44.50)	122 (30.50)
8	I prefer to be prescribed a well-known brand.	158 (39.50)	131 (32.75)	99 (24.75)	12 (3.00)
9	I prefer to be prescribed imported rather than local medicines.	150 (37.50)	143 (35.75)	87 (21.75)	20 (5.00)
10	Costs should be considered before a drug is prescribed.	3 (0.75)	81 (20.25)	220 (55.00)	96 (24.00)
11	I don’t mind whether my prescribed / dispensed medicine is locally produced or imported as long as it is effective.	0 (0.00)	85 (21.25)	217 (54.25)	98 (24.50)
12	I prefer to be prescribed / dispensed the cheapest medicine available for the treatment of my condition.	18 (4.5)	14 (3.50)	251(62.75)	117 (29.25)
13	Cost is not an issue for me as long as the medicine will treat my condition.	103 (25.75)	214 (53.50)	41 (10.25)	42 (10.50)
14	A more expensive medicine is a better one.	157 (39.25)	99 (24.75)	69 (17.25)	75 (18.75)
15	Imported medicines are better.	154 (38.50)	127 (31.75)	66 (16.50)	53 (13.25)
16	Using generic medicines would provide significant saving to me.	0 (0.00)	87 (21.75)	229 (57.25)	84 (21.00)
17	In general, medicine costs in Jordan are too high.	3 (0.75)	65 (16.25)	203 (50.75)	129 (32.25)

**Table 3:** Statistically significant correlations calculated using Chi square test between the statements on the left with each of the demography category investigated

	**Survey questions/Statement**	**Demography criteria**			
		***The monthly income***	***Educational level***	***Percentage paid from the cost***	***No. of medicines in the prescription***
		**Chi square value**			
1	Physicians should ask patients about their medicines preference.	NS	158.38**	NS	NS
2	Patients should have the option of choosing between generic and originator.	NS	163.53**	NS	NS
3	I don’t mind the pharmacist substituting the medicine I was prescribed to a cheaper equivalent one.	52.15**	NS	24.00**	42.03**
4	I don’t mind my prescribed medicines to be substituted from originator to generic. (e.g. Panadol to Revanin).	65.12**	NS	45.95**	48.84**
5	My medicines should only be substituted from originator to generic if I request. (e.g. Panadol to Revanin).	146.12**	NS	NS	46.63**
6	I don’t mind the pharmacist substituting my prescribed medicine to an equivalent locally produced one.	NS	NS	NS	NS
7	I prefer to be prescribed locally produced medicines.	66.23**	NS	36.02**	55.220**
8	I prefer to be prescribed a well-known brand.	NS	NS	NS	NS
9	I prefer to be prescribed imported rather than local medicines.	16.73*	NS	16.83*	24.69**
10	Costs should be considered before a drug is prescribed.	13.83*	NS	24.07**	43.41**
11	I don’t mind whether my prescribed / dispensed medicine is locally produced or imported as long as it is effective.	NS	NS	NS	NS
12	I prefer to be prescribed / dispensed the cheapest medicine available for the treatment of my condition.	21.13**	NS	NS	177.45**
13	Cost is not an issue for me as long as the medicine will treat my condition.	22.65**	NS	40.02**	68.48**
14	A more expensive medicine is a better one.	55.06**	NS	NS	142.07**
15	Imported medicines are better.	21.17**	34.72**	29.26**	134.66**
16	Using generic medicines would provide significant saving to me.	13.23*	NS	92.07**	NS
17	In general, medicine costs in Jordan are too high.	28.59**	NS	46.59**	59.87**

*:p < 0.05, **:P <0.01, NS: non statistically significant correlations found.

Most of the respondents (78%, n = 312) agreed that they should have the option of choosing between generic and originator (Table 2). A chi-square statistic found a significant correlation (P < 0.05) between the educational level of the responders and whether or not they should be given the choice between generic or originator (Table 3). Patients with higher education levels tended to agree or strongly agree with being given the choice.

### Perceptions on generic substitution

When patients were asked if they mind the pharmacist substituting their prescribed medicine, 75% responders did not mind the substitution to a cheaper equivalent (n = 300) (Table 2). In addition, most patients (78%, n = 312) did not mind their prescribed originator medicine being substituted to a generic one (Table 2). There was a significant correlation (P < 0.05) between the patients’ monthly income level, percentage cost paid for the prescription and number of medicines in the prescription and whether or not they minded their prescribed medicine to be substituted to a cheaper medicine or a generic. Patients with lower income, pay more percentage of their medicines cost, and are on a higher number of medicines tended to accept the substitution more. The values of chi square are shown in Table 3.

Most responders (63.5%) preferred to accept generic substitution only upon their request (n = 254) (Table 2). There was a significant correlation (P < 0.05) between patients’ income level and number of medicines in the prescription with their preference for generic substitution to be based on their request (Table 3). Patients with high income levels, and who have small numbers of medicines in their prescription, tended to agree or strongly agree with the substitution being upon their request only. However, there was no correlation with percentage paid from medicines cost and the acceptance of generic substitution upon patients’ request. Interestingly, there was no correlation between the education level of the responders and their preference to be consulted prior to originator generic substitution.

### Opinions regarding locally produced generic medicines

When assessing the patients’ views on locally produced generic medicines, 75% of them preferred to be prescribed locally produced medicines (n = 300) and 73.25% patients did not prefer to be prescribed imported rather than local medicines (n = 293) There was a significant correlation (P < 0.05.) between patients’ monthly income level, percentage cost paid for their medicines and number of medicines in the prescription and their preference for local medicines. Patients with low income, or more percentage cost of medicines and have higher number of prescribed medicines tended to agree or strongly agree with being prescribed locally produced medicines (Table 3). Whereas there was no correlation with the education level of responders and their preference for imported products or locally produced products.

When asked if imported medicines are better than locally produced ones, 70.25% of the surveyed patients disagreed (n = 281) (Table 2). Patients with higher education level, lower income level, pay more percentage cost of medicines and have higher numbers of medicines tended to disagree with imported medicines being better than locally produced (P < 0.05) (Table 3).

The majority of patients (72.25%, n = 289) did not prefer to be prescribed a well-known medicine brand with 78.25% agreeing for their medicines to be substituted to a locally produced generic one (n = 313).

In general, the effectiveness of the medicines is the determinant in patients preference not the manufacturer country according to 78.75% of the responders (n = 315) (Table 2).

### Jordanian patients’ opinions regarding cost of the medicines

The majority of the surveyed Jordanian patients (79%, n = 316) agreed that the costs should be considered before a drug is prescribed (Table 2). There was a significant relationship (P < 0.05) between the monthly income of the patient and percentage paid from the cost of medicine and number of medicines in the prescriptions and their agreement. Patients with low income level, who pay more percentage cost of medicines or who have high number of prescribed medicines tended to agree more that costs should be considered before a drug is prescribed.

Patients predominantly (92%, n = 368) preferred to be prescribed and/or dispensed the cheapest medicine available (Table 2). People with low income, high number of medicines in their prescription tended to prefer to be prescribed and/or dispensed the cheapest medicine available for the treatment of their medical condition (P < 0.05) (Table 3). However, there was no significant correlation between the percentage paid from medicines cost and the preference to be prescribed or dispensed the cheapest medicine available.

Most of the patients (79.25, n = 317) disagreed to the statement “cost is not an issue for me as long as the medicine will treat my condition” (Table 2). A Chi-Square test of independence revealed a significant relationship (P < 0.05) between this response and the monthly income of the patient, the% they pay from the cost of their medicines and the number of medicines in their prescription. Patients with low income level, or pay full cost of medicines or are on high numbers of medicines tended to disagree more with the above statement (Table 3).

Most of the patients (64%, n = 256) disagreed that a more expensive medicine is a better one. Patients with low income level or who are on a high numbers of medicines tended to disagree that a more expensive medicine is a better one (P < 0.05) (Table 3). However, there was no significant correlation with the percentage paid from medicine cost or educational level and the response to the above statement.

Patients predominantly (83%, n = 332) believed that the medicine costs in Jordan are too high (Table 2). There was a relationship between the monthly income of the patient, the percentage paid from the cost of medicines and the number of prescribed medicines and the agreement to this statement (P < 0.05) (Table 3). Patients with low income level, or pay more percentage cost of medicines or are on high number of medicines tended to agree more that medicine costs in Jordan are too high.

### Saving from using generic medicines

Most of the Jordanian patients (78.25% n = 313) believed that the use of generic medicines would provide significant saving to them (Table 2). Patients with low income levels, or pay more percentage cost of medicines tended to believe that the use of generic medicines would provide significant saving for them (P < 0.05) (Table 3). However, there was no significant correlation with number of medicines in the prescription and the belief of saving by using generic medicines.

## Discussion

In this study, the majority of patients (83%) believed that the costs of medicines in Jordan are too high. Moreover, the costs of medicines were found to be a significant issue for about 80% of the surveyed Jordanian patients, which in turn might affect their adherence to treatments [22-24,29]. These results were mostly reported by low income patients, patients who pay for medicines, and patients who have high number of medicines in their repeated prescriptions.

In low income countries, the health services are believed to be of a poor quality [30] and many of the insurance schemes do not provide medicines benefits, or do so with substantial co-payments [31]. Therefore, medicines are still mainly purchased through out-of-pocket payments [32]. Results from a study in 36 developing and middle-income countries showed that patients purchasing medicines in private sectors pay on average 2.6 times more for originator brands compared to their generic equivalent [33]. This is considered as a barrier to medicines access [34]. In Jordan it was reported that over 80% of the cost of medicines purchased by the public is funded through out-of pocket payments 26 This was reflected in the population of this study, where about 80% of the surveyed patients either paid full or part costs of their medicines.

In the current survey, just under than 80% of the respondents agreed that costs should be considered before a drug is prescribed. In addition, Jordanian patients surveyed predominantly (92%) preferred to be prescribed and/or dispensed the cheapest medicine available for the treatment of their medical condition. Furthermore, the results showed the high trust and confidence of Jordanian patients in locally produced generic medicines. More than third of the respondents preferred to be prescribed a cheaper locally produced generic medicine rather than a more expensive imported brand medicine. Overall, almost 80% of the patients believed that the use of generic medicines would provide significant saving to them.

Most patients (78%) accepted their prescribed originator medicine being substituted to a generic one. With 75% and 78% accepting the pharmacist substituting their medicines to a cheaper one or to locally produced generic one respectively. This was almost the same result of a previous study that was held in Australia where 78.5% of the patients accepted generic substitution based on pharmacists’ recommendation [35]. Another study in New Jersey, USA reported that 97% of the patients who had been offered substitution had agreed to switch their therapy [19]. This also corresponds to a study in Finland in which 81% of the participants were of the opinion that cheaper generics were effective and 85% did not consider generics substitution as a threat to drug safety [36]. On the other hand, a Slovakian study reported that only 50% indicated a preference for a cheaper product [14].

In America, 66.7% of the patients requested substitution to generic medications from doctors or pharmacists in most or all time [37]. However, 63.5% of responders in Jordan accepted generic substitution only upon their request, those respondents were mainly the patients with high monthly income, and/or have less number of medicines in their repeated prescription and /or have a full medical insurance. This would indicate that these groups of patients are less sensitive to the cost of medications.

This study found that patients, generally, have acceptability to generic substitution, consistent with previous studies in Denmark, Spain and Norway where preference for the use of generics among patients was reported [38,39].

It was reported that patients’ communication with physicians has a key role to promote the use of generic medicines, as their preferences are a powerful motivator to the physicians’ prescribing behaviour [40-42]. However, patients hardly ever communicate with their physicians about medication choices and out-of-pocket costs of medications [15,43]. Almost third of the patients in this study believed that they should be involved on decisions regarding their medicines preference, and to have the option of choosing between generic and originator. These beliefs were reported mainly by highly educated participants, similar findings were reported in two different studies in Sweden, in the first study higher educated respondents were 8 times more likely to be involved in choosing and deciding the alternative medicines if available [44]. In the second one, 94% of the patients wanted some involvement in medicine decision making, with positive association between education and shared decision making [45]. Moreover, it is believed that patients who are involved in their medicines decision are more likely to adhere to their treatment with concomitant improvement in health [46].

Physicians’ prescribing behaviour can also be influenced by pharmaceutical companies through a variety of incentives such as high-end education programs or even some cash payment for prescriptions [47]. These incentives may indirectly affect the patients, by encouraging them to use higher priced originator-branded products instead of equally effective, lower-cost generics [48]. Therefore, it has generally been agreed that patients should be involved in decisions making about their own health and treatment all over the world [49,50]. Therefore, The Professional Medical Body in Jordan should develop good practice standards that require clinicians to involve patients in treatment choices. This could be through well-designed training courses that improve the communication skills of doctors, nurses and pharmacists with patients.

From this study, it is clearly obvious that Jordanian patients have a positive attitude towards generic medicines, locally produced medicines, generic substitution, and that they prefer to be involved in medicine treatment selection. This would facilitate the introduction of a generic policy in Jordan which encourages the utilisation of generic medicines through generic substitution and generic prescribing. As a result a huge saving could be achieved to both patients and the health care system.

## Conclusion

The high cost of medicines in Jordan is believed to be the main driver for choosing generic medicines which would lead to substantial saving as identified by the findings. Furthermore, patients have positive attitudes towards generic medicines in general and locally produced ones in particular. The involvement of patients in the treatment decision making allow them to choose the preferred medicine, this would result in more adherence and improvement in health.

The insights gained from patients in this study will be useful to health organisations and policy makers to design a robust generic policy to use medicines cost-effectively in Jordan.
